# Ultrasound assisted aqueous two-phase extraction of polysaccharides from *Cornus officinalis* fruit: Modeling, optimization, purification, and characterization

**DOI:** 10.1016/j.ultsonch.2022.105966

**Published:** 2022-03-01

**Authors:** Jiaqi Tan, Pengshan Cui, Shaoqin Ge, Xu Cai, Qian Li, Hongkun Xue

**Affiliations:** a College of Traditional Chinese Medicine, Hebei University, No. 342 Yuhua East Road, Lianchi District, Baoding 071002, China; b College of Quality and Technical Supervision, Hebei University, No. 2666 Qiyi East Road, Lianchi District, Baoding 071002, China; c Key Laboratory of Particle & Radiation Imaging, Ministry of Education, Department of Engineering Physics, Tsinghua University, No. 30 Shuangqing Road, Haidian District, Beijing 100084, China

**Keywords:** *Cornus officinalis*, Ultrasound assisted aqueous two-phase extraction, Polysaccharides, Optimization, Characterization

## Abstract

Ultrasound assisted aqueous two-phase extraction of polysaccharides from *Cornus officinalis* fruit was modeled by response surface methodology (RSM) and artificial neural network (ANN), and optimized using genetic algorithm coupled with ANN (GA-ANN). Statistical analysis showed that the models obtained by RSM and ANN could accurately predict the *Cornus officinalis* polysaccharides (COPs) yield. However, ANN prediction was more accurate than RSM. The optimum extraction parameters to achieve the highest COPs yield (7.85 ± 0.09)% was obtained at the ultrasound power of 350 W, extraction temperature of 51 ℃, liquid-to-solid ratio of 17 mL/g, and extraction time of 38 min. Subsequently, the crude COPs were further purified via DEAE-52 and Sephadex G-100 chromatography to obtain a homogenous fraction (COPs-4-SG, 33.64 kDa) that contained galacturonic acid, arabinose, mannose, glucose, and galactose in a molar ratio of 34.82:14.19:6.75:13.48:12.26. The structure of COPs-4-SG was also characterized with UV–vis, fourier-transform infrared spectroscopy (FT–IR), atomic force microscopy (AFM), scanning electron microscopy (SEM), Congo-red test, and circular dichroism (CD). The findings provide a feasible way for the extraction, purification, and optimization of polysaccharides from plant resources

## 1 Introduction

*Cornus officinalis*, as a traditional precious Chinese herbal medicine in China, is widely cultivated in Henan, Zhejiang, Shanxi, Hunan, and other provinces in China. The fruit of *Cornus officinalis* is rich in iridoid glycosides, flavonoids, polyphenols, tannins, polysaccharides, and other active components [1], and the polysaccharides are one of the most abundant active components in *Cornus officinalis* fruit. Mounting evidences have indicated that *Cornus officinalis* polysaccharides (COPs) have many bioactivities, such as antioxidant, anti-tumor, anti-inflammatory, anti-microbial, and anti-thrombotic activities [2], [3]. The extraction of polysaccharides is the most critical step for its development or application. Nevertheless, little attention has been paid to the extraction and purification of COPs. Consequently, an efficient extraction method and optimize the extraction process must be investigated to obtain the higher COPs yield.

Currently, many techniques for extraction polysaccharides from natural plant resources include hot water extraction (HWE)[4], supercritical fluid extraction (SFE)[5], ultrasound assisted extraction (UAE)[6], and microwave assisted extraction (MAE)[7]. HWE is the most commonly used extraction method of polysaccharides from natural resources that has many merits including simple operation, no special equipment required, and easy implementation. However, long extraction time and high temperature may destroy the structure of polysaccharides and reduce its biological activities [4]. SFE parameters are difficult to control, complex operation, and high equipment requirements, which limit the large-scale popularization of SFE technology[5]. MAE is a promising extraction method because of its high extraction efficiency and easy process control. Nevertheless, the local high temperature of the extract caused by microwave radiation may cause the degradation of polysaccharides, which is not conducive to the extraction of polysaccharides [7]. Among these extraction techniques, UAE is a novel extraction method that can obtain target active ingredient with the lower solvent consumption and production cost, faster rate, and higher recovery compared with conventional HWE[8]. Ultrasound produces cavitation effect, mechanical effect, and thermal effect in the solvent, which makes the solvent molecules penetrate into the material matrix faster to accelerate the release of target compounds in the solvent [9]. Therefore, UAE is extensively employed to extract active components from different plants. However, the above extraction methods used a single extraction solvent to extract plant polysaccharides, resulting in relatively low yield of polysaccharides.

Recently, the application scope of aqueous two-phase extraction (ATPE) has been gradually expanded due to its high yield, friendly environment, easy amplification, low cost, and little damage to molecular biological activity [10]. As a new extraction solvent, aqueous two-phase extraction system (ATPS) can extract and purify the various compounds from natural plant resources in one step [11]. Especially, short chain alcohol aqueous salt solution has been extensively used in the extraction of active components owing to its advantages of easy separation, low viscosity, and solvent recycling [12]. The combined method of UAE and ATPE, namely, ultrasound assisted aqueous two-phase extraction (UAATPE), has been used to extract some effective components from herbal materials [13], [14], [15]. This method completes the extraction and purification in one step. In this way, the polysaccharides components were optionally migrated to the bottom phase, whereas the most impurities are extracted to the upper phase. Therefore, UAATPE is one of the most suitable extraction methods for polysaccharides from natural plant. To data, the study on UAATPE polysaccharides from *Cornus officinalis* fruit has not been reported. Many experimental parameters in the extraction process will affect the yield of active compounds from natural plant sources. Hence, it is necessary to optimize the extraction parameters to obtain the maximum COPs yield. Response surface methodology (RSM) or artificial neural network (ANN) can be employed for process modeling to predict the yield of target components under different extraction conditions. RSM, as a collection of statistical method, is employed to design experiment, develop model, and evaluate the impact of processing parameters on responses [16]. ANN is employed to model complex biological processes and highly non-linear results, which overcomes the problems difficult to be solved by artificial or statistical methods [17]. Many researches have confirmed that ANN was more popular than conventional regression models for optimization and prediction [18], [19]. In addition, ANN is superior to RSM, unpredictable nonlinear data, fuzzy input, and subtle mode.

Genetic algorithm (GA) is a population optimization algorithm without mathematical model, which realizes random, adaptive, and global optimization based on natural selection theory and genetic principle. The GA coupled with ANN (GA-ANN) has been used to optimize the extraction process of various active components [20], [21], [22]. However, the optimization UAATPE process of polysaccharides from *Cornus officinalis* fruit by GA-ANN has not been reported. In addition, the isolation, purification, and structural characterization of COPs obtained by UAATPE have not also been reported yet. Therefore, this study aimed to model by RSM and ANN methods, and optimize the extraction process of UAATPE using GA-ANN. Furthermore, the crude COPs were purified by DEAE-52 and Sephadex G-100 chromatography to finally obtain a homogeneous polysaccharides (COPs-4-SG), which was characterized by UV–vis, FT-IR, AFM, SEM, Congo-red test, and CD.

## 2 Materials and methods

### 2.1 Material and chemicals

The fruit of *Cornus officinalis* was offered from Yuelin Pharmaceutical Co., Ltd (Bozhou, China). DEAE-52 cellulose was from Solabao Technology Co., Ltd (Beijing, China). Sephadex G-100 was provided from Ruji Biotechnology Development Co., Ltd (Shanghai, China). Monosaccharide standards (arabinose, xylose, mannose, galacturonic acid, rhamnose, fucose, glucose, and galactose) were obtained from Chengshao Biotechnology Co., Ltd (Shanghai, China). Phenol, ammonium sulfate, sulphuric acid, sodium chloride, methanol, and ethanol (analytical purity) were obtained from Kaisai Chemical Co., Ltd (Shanghai, China).

### 2.2 Preparation of sample and ATPS

The fruit of *Cornus officinalis* was dried by a vacuum oven (TY-2 K-1, Taiyu oven equipment Co., Ltd, Suzhou, China) at 50 °C for 48 h, and then passed a small plant crusher (JJ-600, Tairi Machinery Technology Co., Ltd, Guangzhou, China) to obtain the powder samples for subsequent experiments.

The ATPS of ethanol/(NH_4_)_2_SO_4_ system was prepared based on the reported phase chart [23]. The ATPS (25.4% ethanol-22.2% (NH_4_)_2_SO_4_) was obtained when the mixture presented upper and lower phase separation.

### 2.3 UAATPE procedure

1.0 g of the *Cornus officinalis* powder was dissolved in 60 mL of ATPS, and then fully oscillated, sealed, and placed in an ultrasound equipment. According to the previous experimental results in the laboratory, the ultrasound power, extraction temperature, and ultrasound time were selected as 400 W, 50 °C, and 30 min, respectively. The filtrates were combined and centrifuged (5000 g for 15 min) by using a bench type high speed centrifuge, and then the lower phase and the upper phase were collected, respectively. Subsequently, the lower phase was used to determine the COPs yield.

### 2.4 Experimental design

Box-Behnken design (BBD) based on RSM with four experimental factors was employed to design the experiments in this study. The experimental variables included ultrasound power (*X*_1_), extraction temperature (*X*_2_), liquid-to-solid ratio (*X*_3_), and extraction time (*X*_4_). According to the preliminary study conducted in the laboratory, the input parameters and their ranges are set. The ultrasound power (*X*_1_, 200–400 W), extraction temperature (*X*_2_, 50–70 °C), liquid-to-solid ratio (*X*_3_, 15–25 mL/g), and extraction time (*X*_4_, 20–40 min) were selected as independent experimental variables. A total of 30 trials was carried out in a randomized order to minimize the impact of external factors (Table 1).

**Table 1:** Experimental design and results of RSM.

No.	Variable				COPs yield/%
	*X*_1_ /W	*X*_2_ /℃	*X*_3_ /(mL/g)	*X*_4_ /min	
1	−1 (2 0 0)	−1 (50)	0 (20)	0 (30)	6.95
2	1 (4 0 0)	−1 (50)	0 (20)	0 (30)	7.03
3	−1 (2 0 0)	1 (70)	0 (20)	0 (30)	7.24
4	1 (4 0 0)	1 (70)	0 (20)	0 (30)	7.21
5	0 (3 0 0)	0 (60)	−1 (15)	−1 (20)	6.93
6	0 (3 0 0)	0 (60)	1 (25)	−1 (20)	7.16
7	0 (3 0 0)	0 (60)	−1 (15)	1 (40)	7.11
8	0 (3 0 0)	0 (60)	1 (25)	1 (40)	7.21
9	−1 (2 0 0)	0 (60)	0 (20)	−1 (20)	7.13
10	1 (4 0 0)	0 (60)	0 (20)	−1 (20)	6.89
11	−1 (2 0 0)	0 (60)	0 (20)	1 (40)	6.99
12	1 (4 0 0)	0 (60)	0 (20)	1 (40)	7.28
13	0 (3 0 0)	−1 (50)	−1 (15)	0 (30)	6.83
14	0 (3 0 0)	1 (70)	−1 (15)	0 (30)	7.38
15	0 (3 0 0)	−1 (50)	1 (25)	0 (30)	7.26
16	0 (3 0 0)	1 (70)	1 (25)	0 (30)	7.23
17	−1 (2 0 0)	0 (60)	−1 (15)	0 (30)	6.92
18	1 (4 0 0)	0 (60)	−1 (15)	0 (30)	7.06
19	−1 (2 0 0)	0 (60)	1 (25)	0 (30)	7.11
20	1 (4 0 0)	0 (60)	1 (25)	0 (30)	7.19
21	0 (3 0 0)	−1 (50)	0 (20)	−1 (20)	6.97
22	0 (3 0 0)	1 (70)	0 (20)	−1 (20)	7.16
23	0 (3 0 0)	−1 (50)	0 (20)	1 (40)	6.93
24	0 (3 0 0)	1 (70)	0 (20)	1 (40)	7.31
25	0 (3 0 0)	0 (60)	0 (20)	0 (30)	7.42
26	0 (3 0 0)	0 (60)	0 (20)	0 (30)	7.39
27	0 (3 0 0)	0 (60)	0 (20)	0 (30)	7.31
28	0 (3 0 0)	0 (60)	0 (20)	0 (30)	7.50
29	0 (3 0 0)	0 (60)	0 (20)	0 (30)	7.16
30	0 (3 0 0)	0 (60)	0 (20)	0 (30)	7.24

### 2.5 Polysaccharides yield

The COPs yield was determined referring to the report of Sun et al. (2019) with slight modification. The COPs yield (*Y*) was calculated by equation (1) [24].

(1) $$Y=\frac{\mathrm{Mass}\;of\;extracted\;polysacchaides}{\mathrm{Mass}\;of\;Cornusofficinalisfruitpowder\;}\times 100$$

### 2.6 RSM model

The relationship between experimental variables and COPs yield was analyzed by Design Expert version 8. The experimental results were fitted by equation (2) to obtain the model regression coefficient.

(2) $$Y=\beta_{0}+\sum_{j=1}^{k}\beta_{j}X_{j}+\sum_{j=1}^{k}\beta_{\mathrm{jj}}X_{j}^{2}+\sum_{i}\sum_{<j=2}^{k}\beta_{\mathrm{ij}}X_{i}X_{j}+e_{i}$$

where, *Y* is the COPs yield, %; *X_i_* and *X_j_* are the coded variables (*i* and *j* range from 1 to *k*); *β*_0_, *β_j_*, *β_jj_*, and *β_ij_* are regression coefficients of intercept coefficient, linear, quadratic and the second-order terms, respectively; *k* is the number of independent parameters (*k* = 4) and *e_i_* is the error.

The analysis of variance (ANOVA) was used to analyze the RSM model. The coefficient of determination (*R*^2^) and lack-of-fit were calculated to determine the adequacy of RSM model, and the relative dispersion of the experimental points was measured by calculating the coefficient of variation (C.V.).

### 2.7 ANN model

**Fig. 1:**
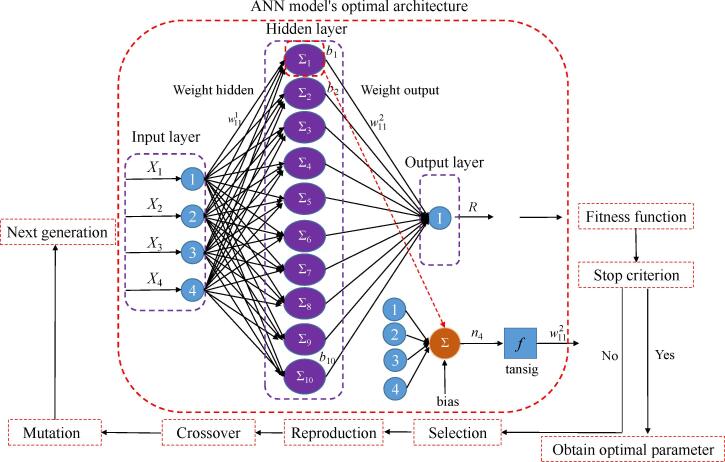
Optimal architecture of the ANN and GA optimization steps, post-training.

The neural network fitting tool of MATLAB was employed for modelling of experimental results through ANN that was produced during UAATPE polysaccharides from *Cornus officinalis* fruit. Fig. 1 shows the ANN model structure with independent and dependent variables. ANN structure consists of input layer (*X*_1_, *X*_2_, *X*_3_, and *X*_4_), hidden layer, and output layer (COPs yield). The neural network model was trained until the error achieved the minimum values between the experimental value and the predicted value of COPs yield. The experimental data was trained by Levenberg-Marquardt back propagation algorithm (trainlm) because it is the fastest and most accurate algorithm in the toolbox. The weight and deviation are called as neural network parameters. The “trainlm” randomly divides three subsets: training, validation, and testing with 80% for training, 10% for validation, and 10% for testing. The transfer functions of the hidden layer and the output layer are the hyperbolic tangent sigmoid function (tansig) and linear function (purelin), respectively. All experiment results were normalized between −1 and 1 by equation (3) [25]. These standardized values are converted into actual values after passing through the output layer of the network.

(3) $$M_{i}=\frac{\left(M_{\max}-M_{\min}\right)\left(N_{i}-N_{\min}\right)}{N_{\max}-N_{\min}}+N_{\min}$$

where, *M_i_* is the normalized value. *M*_max_ and *M*_min_ are the maximum and the minimum values of the scaling range. *N_i_* is the actual data to be normalized. *N*_max_ and *N*_min_ are the maximum and minimum values of the actual data.

After ANN modeling, the trained ANN was transformed into a mathematical equation through weight, deviation, and transfer function:

$$Y\left(\%\right)=purelin\left(\left[\sum_{i=1}^{N}w_{i}^{2}\text{tansig}\left(\sum_{j=1}^{j}w_{i,j}^{1}x_{j}+b_{1i}\right)\right]+b_{2}\right)$$

(4) $$\text{tansig}\;\left(x\right)=\frac{2}{1+\exp\;(-2x)}-1$$

$$\mathrm{purelin}\;\left(\text{x}\right)=x$$

where, *x* and *j* are the experimental factors (the input variables) and the number of input variables, respectively. *w*^1^ and *b*_1_ are the weight and bias of hidden layer, respectively. *w*^2^ and *b*_2_ are the weight and bias of output layer, respectively.

### 2.8 Analysis of the developed models

The values of *R*^2^, mean squared error (*MSE*), root mean squared error (*RMSE*), sum of squares dueto error (*SSE*), akaike information criterion (*AIC*), and absolute average deviation (*AAD*) were used to evaluate the prediction performance of RSM and ANN [26].

(5) $$R^{2}=1-\frac{\sum_{i=1}^{n}{\left(x_{i}-x_{\mathrm{ik}}\right)}^{2}}{\sum_{i=1}^{n}{\left(x_{\mathrm{ik}}-x_{z}\right)}^{2}}$$

(6) $$\mathrm{MSE}=\frac{1}{n}\sum_{i=1}^{n}{\left(x_{i}-x_{\mathrm{ik}}\right)}^{2}$$

(7) $$\mathrm{RMSE}=\sqrt{\frac{1}{n}\sum_{i=1}^{n}{\left(x_{i}-x_{\mathrm{ik}}\right)}^{2}}$$

(8) $$\mathrm{SSE}=\frac{1}{n^{2}}\sum_{i=1}^{n}{\left(x_{i}-x_{\mathrm{ik}}\right)}^{2}$$

(9) AIC=nln(SSE)+2p

(10) $$\mathrm{ADD}\;\left(\%\right)=\left[\frac{\sum_{i=1}^{n}\left(\left|x_{\mathrm{ik}}-x_{i}\right|/x_{\mathrm{ik}}\right)}{n}\right]\times 100$$

where, *x_i_* is predicted COPs yield. *x_ik_* is the experimental or actual COPs yield. *x_z_* is the mean of experimental COPs yield. *n* and *p* represent the number of data points and parameters used in each model, respectively.

### 2.9 Optimization of the process

GA coupled with the developed ANN optimized the extraction parameters of COPs. The purpose of GA was to generate the maximum values, and transformed the minimization problem into the maximization problem by changing the fitness values. It is realized by converting a function into an inverse function or by changing the sign. The natural phenomena such as species reproduction, crossover, mutation, and selection are simulated for GA optimization through the GA toolbox of MATLAB. The ANN-derived equation (4) was introduced as a fitness function. The higher the COPs yield, the greater the individual fitness values. The values of initial population size, crossover fraction, mutation fraction, and evolutionary algebra are selected according to the actual situation, and the default values of other parameters are selected.

### 2.10 Purification

The extract in the lower phase of crude polysaccharides from *Cornus officinalis* fruit obtained under the optimal extraction parameters was concentrated by a rotary evaporator (RE-3000A, Xiren Scientific Instrument Co., Ltd, Shanghai, China), and the concentrated solution was precipitated with 80% ethanol overnight. .The lyophilized sample was dissolved with deionized water and centrifuged to obtain the supernatant, and it was eluted with deionized water and different concentrations of NaCl (0.1, 0.2, 0.3, 0.4, and 0.5 mol/L) solution on the DEAE-52 cellulose column at 1.0 mL/min. Subsequently, the eluent was continuously collected into test tubes by an automatic fractionator, and each test tube contained 5 mL eluent. The content of polysaccharides was determined by the phenol–sulfuric acid method (PSAE). Five polysaccharides fractions (COPs-1, COPs-2, COPs-3, COPs-4, and COPs-5) were obtained after separation by the above method, and the polysaccharides content of COPs-4 was the highest. Next, the main fraction COPs-4 was further separated and purified by Sephadex G-100 column (1.6 cm × 50 cm) using deionized water. Elution parameters are as follows: The loading concentration of 10 mg/mL, the loading volume of 4 mL, and the flow rate of 0.4 mL/min. Each tube collected 2 mL fraction, and it was measured again by the PSAE. The purified polysaccharides fraction was freeze-dried to obtain COPs-4-SG for further analysis.

### 2.11 Characterization

#### 2.11.1 UV-vis

This study referred to the method described by Kia, Ganjloo, & Bimakr, (2018) with slight modifications [27]. In short, COPs-4-SG was prepared into sample solution with appropriate concentration, and the sample solution was scanned by an UV spectrophotometer (UV-5600, Puyuan Instrument Co., Ltd, Shanghai, China) in the wavelength range of 200–800 nm to verify whether the sample solution contained protein.

#### 2.11.2 Monosaccharide composition

The monosaccharide composition of COPs-4-SG was measured based on the description of Hui et al. (2019) with slight modifications [28]. Briefly, COPs-4-SG was completely hydrolyzed to monosaccharide with trifluoroacetic acid, after derivatization with saccharin acetyl, the gas chromatography (GC-2010 plus, Shimadzu Corporation, Japan) with monosaccharides standards was used to detect the monosaccharide derivatives.

#### 2.11.3 Determination of molecular weight

COPs-4-SG was dissolved in deionized water at 1 mg/mL, filtered through a 0.45 μm membranes, and then injected into the high performance gel permeation chromatography (HPGPC). The HPLC system was equipped with a refractive index detector (RID) and a ultra-hydrogel linear gel filtration column. The elution was performed with deionized water. The calibration curve was constructed using several dextran standards to measure the average molecular weight (M_W_) [29]. The empower software was used to analyze the experimental data.

#### 2.11.4 FT–IR

The FT–IR spectra of COPs-4-SG were obtained by using a FT–IR spectrometer. The sample (1 mg) was mixed with KBr in a 1:30 (w/w) ratio and compressed into slices. The scanning was carried out with a wavelength range of 4000–400 cm^−1^[30]. The spectra was analyzed by the spectrophotometer’s built-in software.

#### 2.11.5 SEm

The microstructure of COPs-4-SG was taken via a SEM (JSM-7401, Japan Electronics Corporation, Tokyo, Japan). Briefly, the sample powder was coated with gold for 5 min to perform the test at 5.0 kV[31]. To assure clear micrographs, the XT Microscope Control software was used to obtain digitally all micrographs.

#### 2.11.6 AFM

COPs-4-SG was prepared with distilled water with a concentration of 10 μg/mL sample solution, and then it was passed through 0.22 μm membranes. 10 μL of COPs-4-SG sample solution was dropped on the surface of mica sheet and dried overnight at 25 °C. The molecular morphology of polysaccharides was observed by using a Multimode 8 AFM (Brooke Corporation, USA)[32]. The other parameters are set as follows: The mechanical constant for silicon cantilever and the radius of curvature of the probe tip were 12.715 N/m and 8.53 μm, respectively.

#### 2.11.7 Congo-red

The Congo red method was employed to confirm the triple helix conformation of COPs-4-SG based on the method described by Liu et al. (2016) [33]. In short, COPs-4-SG (2.0 mL, 2.5 mg/mL) was mixed with 2.0 mL of Congo-red solution (100 μmol/L), and then gradually added 1 mol/L NaOH solution to the mixtures to make the final NaOH concentration 0, 0.1, 0.2, 0.3, 0.4, and 0.5 mol/L. The UV–vis absorption spectrum of COPs-4-SG at 400–700 nm was recorded by an UV spectrophotometer.

#### 2.11.8 Circular dichroism (CD) spectroscopy analysis

COPs-4-SG was dissolve in water at 1.0 mg/mL and analyzed via the CD Spectroscope (J-810, Jasco, Japan) at wavelengths of 190–300 nm under fixed experimental conditions, while the scanning rate is 50 nm/min.

## 3 Results and discussion

### 3.1 RSM modeling

Extraction of polysaccharides from *Cornus officinalis* fruit was studied with the application of BBD based on RSM with four independent variables, namely, ultrasound power (*X*_1_), extraction temperature (*X*_2_), liquid-to-solid ratio (*X*_3_), and extraction time (*X*_4_) along with COPs yield as dependent variables. Table 1 shows the experimental design of BBD and the experimental values under different combinations of four independent variables, and Table 2 represents the response (COPs yield) of ANOVA for all the dependent variables. The model significance was analyzed by using ANOVA. The values of *p* and *F* were used to evaluate the importance of each variable. Low *p* values and high *F* values indicated that the relevant variables are very significant. The results displayed that the model of COPs yield was highly significant at a level of *p* < 0.0001, whereas the lack of fit was not significant (*p =* 0.9985 > 0.005). In the case of COPs yield, *X*_2_, *X*_3_, *X*_1_^2^, *X*_3_^2^, *X*_4_^2^, *X*_1_*X*_4_, and *X*_2_*X*_3_ model parameters were found extremely remarkable at a level of *p* < 0.001, and *X*_4_ and *X*_2_^2^ were prominent at a level of *p* < 0.05, whereas other variables had no marked effect on the COPs yield at a level of *p* > 0.05. In addition, the values of *R*^2^ and C.V. were 0.8906 and 0.7595, respectively.

**Table 2:** ANVOA analysis for RSM model.

Source of variance	*SQ*	*df*	*MS*	*F* value	*p* value
Model	0.77	14	0.055	8.72	< 0.0001**
*X*_1_	8.533E-03	1	8.533E-03	1.36	0.2621
*X*_2_	0.20	1	0.20	32.27	< 0.0001**
*X*_3_	0.072	1	0.072	11.74	0.0041**
*X*_4_	0.029	1	0.029	4.62	0.0484*
*X*_1_^2^	0.15	1	0.15	23.47	0.0002**
*X*_2_^2^	0.0049	1	0.0049	7.73	0.0140*
*X*_3_^2^	0.066	1	0.066	10.46	0.0056**
*X*_4_^2^	0.13	1	0.13	20.75	0.0004**
*X*_1_*X*_2_	3.025E-03	1	3.025E-03	0.48	0.4984
*X*_1_*X*_3_	9.000E-04	1	9.000E-04	0.14	0.7104
*X*_1_*X*_4_	0.070	1	0.070	11.17	0.0045**
*X*_2_*X*_3_	0.084	1	0.084	13.38	0.0023**
*X*_2_*X*_4_	9.025E-03	1	9.025E-03	1.44	0.2494
*X*_3_*X*_4_	4.225E-03	1	4.225E-03	0.67	0.4251
Residual	0.094	15	6.285*E*-0.003		
Lack of fit	0.017	10	1.654*E*-0.003	0.11	0.9985
Pure error	0.078	5	0.016		
Sum	0.86	29			
	*R*^2^ = 0.8906		*R*^2^_Adj_ = 0.7885	*C.V*. = 0.7595	

The insignificant factors were preliminarily removed, and the experimental results were analyzed by multiple regression based on the results of RSM. The regression model of COPs yield was developed in terms of coded values for the experimental factors:

(11) $$\begin{array}{c} Y=7.34+0.13X_{2}+0.078X_{3}+0.049X_{4}+0.13X_{1}X_{4}\;\text{-}\;0.14X_{2}X_{3}\\ -0.15X_{1}^{2}-0.084X_{2}^{2}-0.098X_{3}^{2}-0.14X_{4}^{2} \end{array}$$

The adjusted *R*^2^_adj_ was close to *R*^2^ of the regression model of COPs yield. Moreover, non-significant lack of fit showed that the established regression model is more suitable for the predicted COPs yield under different parameters combinations.

### 3.2 Effect of extraction parameters on COPs yield

**Fig. 2:**
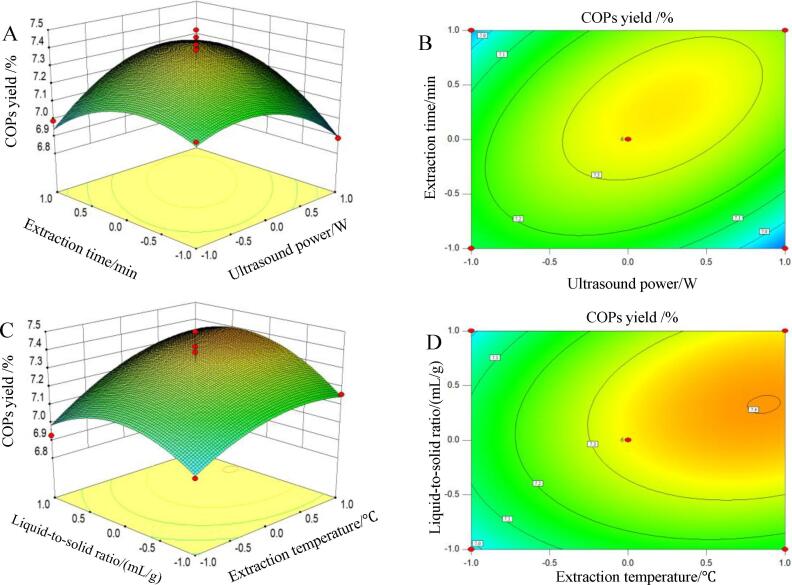
Effect of interaction of two factors on COPs yield: 3D response surface charts (A and C) and corresponding 2D contour curves (B and D).

The 3D response surface and 2D contour are plotted according to equation (9). Fig. 2A displays the COPs yield as a function of ultrasound power (*X*_1_) and extraction time (*X*_4_) when extraction temperature (*X*_2_) and liquid-to-solid ratio (*X*_3_) were set at a zero level. An extremely notable interaction (*p =* 0.0045 < 0.01) was observed between ultrasound power (*X*_1_) and extraction time (*X*_4_) (Table 2). The COPs yield initially increased, and then decreased with the increase of ultrasound power (*X*_1_) and extraction time (*X*_4_). The interaction of caused by ultrasound power (*X*_1_) and extraction time (*X*_4_) in this study is consistent with Gu et al (2020) studying on ultrasound assisted extraction polysaccharides from *Sagittaria sagittifolia* L [34]. Fig. 2C shows that the COPs yield was affect by both extraction temperature (*X*_2_) and liquid-to-solid ratio (*X*_3_). Additionally, Table 2 shows a highly prominent interaction between these variables at *p* = 0.0023 (*p* < 0.01). The COPs yield also initially increased and reached the maximum with the increase of extraction temperature (*X*_2_) and liquid-to-solid ratio (*X*_3_), and the COPs yield appeared a negative response when the extraction temperature (*X*_2_) and liquid-to-solid ratio (*X*_3_) further increased. The similar results were observed by other authors in the case of polysaccharides from common mullein (*Verbascum thapsus* L.) flowers and *Sagittaria sagittifolia* L. by UAE [35], [36]. Fig. 2B and 2D were oval, further suggesting that the interaction of ultrasound power (*X*_1_) and extraction time (*X*_4_), extraction temperature (*X*_2_) and liquid-to-solid ratio (*X*_3_) significantly affected the COPs yield. The results were consistent with the results of ANOVA (Table 2).

### 3.3 ANN modeling

The relationship between the four inputs and the output variable was simulate by ANN. Fig. 1 presents the whole process of ANN modeling. The network parameters are trained and verified to show the robustness of the established network and test the error in the network. The trial and error method was used to determine the number of neurons in the hidden layer the minimum *MSE* was obtained. By analyzing the *MSE* data of neurons in the hidden layer, this study finally determined that the number of neurons in the hidden layer was 10, which was attributed to the minimum *MSE* at this point (Fig. S1). The regression R values were determined by the correlation between the outputs and the targets. The R value of 1 and lower *MSE* values indicated that the outputs are closely related to the targets. Hence, the ANN topology of 4-10-1 was the best topology to optimize the COPs yield (Fig. 1). Each neuron has weights (*w*) and bias (b) from input layer to hidden layer and from hidden layer to output layer, and the resulting structure created a network. The size of weight matrix of the input layer connected to the hidden layer was 10 × 4, and the size of weight matrix of the hidden layer connected to the output layer was 10 × 1, whereas the biases matrix sizes of hidden layer neurons and output layer neurons were 10 × 1 and 1, respectively. The Eqs. (12), (13), (14), (15) were uesd to calculate the ANN parameters.

(12) $$w^{1}=\left[\begin{array}{cccc} 0.343017 & 0.022306 & -1.142627 & 2.184996\\ -0.14592 & 1.904915 & 1.374795 & -0.81118\\ -0.97414 & 1.596739 & -0.55674 & 1.545828\\ -0.52483 & -1.50296 & 1.774247 & -0.71819\\ 0.070061 & -2.20561 & -0.84862 & -0.77992\\ 0.961495 & 1.638237 & -0.48877 & -1.53326\\ -0.93004 & 1.370738 & -1.38043 & 1.24442\\ -1.55736 & 0.303836 & 1.34670 & -1.36630\\ 0.669556 & 1.987972 & -0.72176 & -1.12995\\ -0.266223 & -0.5287 & -1.04532 & -2.18059 \end{array}\right]$$

(13) $$b^{1}=\left[\begin{array}{c} -2.48959\\ 1.936349\\ 1.383106\\ 0.829864\\ -0.27662\\ 0.276621\\ -0.82986\\ -1.38311\\ 1.936349\\ -2.48959 \end{array}\right]$$

(14) $$w^{2}=\left[\begin{array}{c} 0.968797\\ -0.91973\\ -0.57294\\ -0.03547\\ -0.20334\\ -0.10741\\ -0.98684\\ 0.321522\\ -0.31226\\ -0.60018 \end{array}\right]$$

(15) $$b^{2}=\left[0.684928\right]$$

**Fig. 3:**
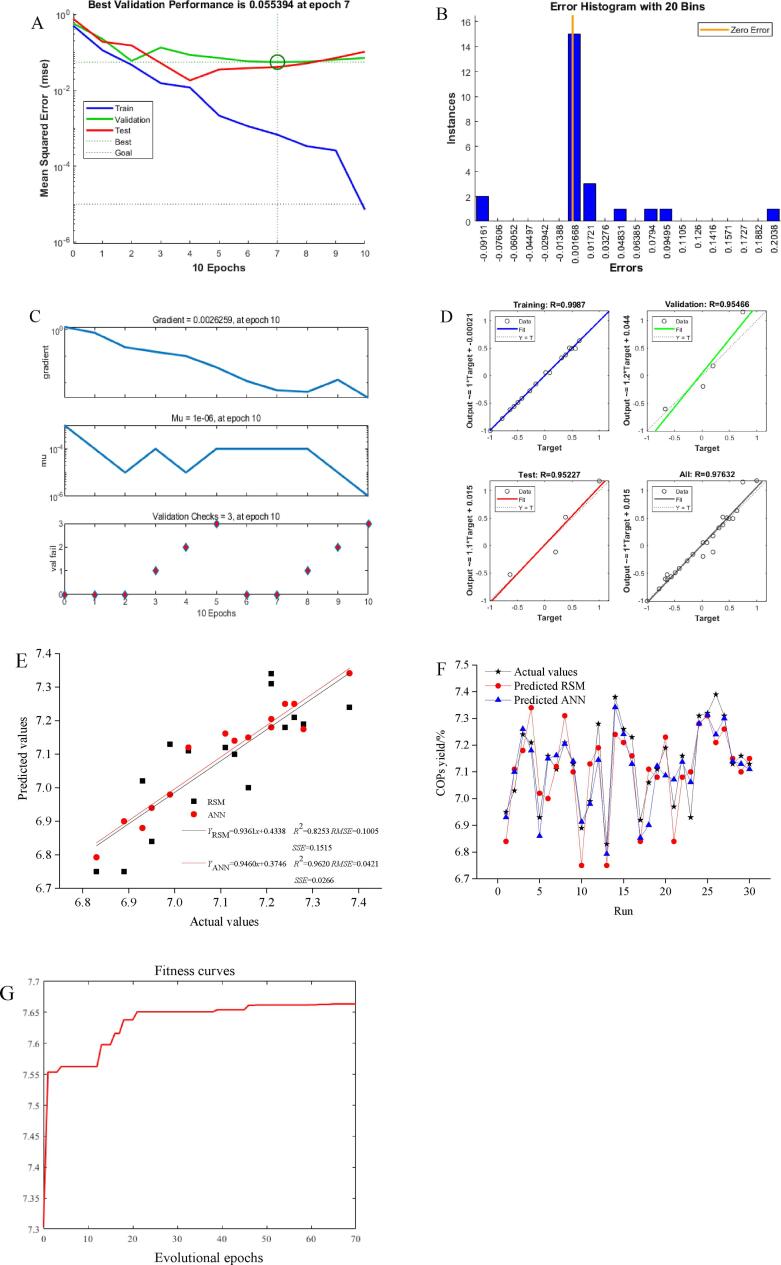
The results of Levenberg–Marquardt algorithm display best validation performance (A), error histogram (B), training state (C), and regression plot (D), relationship between the actual values vs. predicted values by RSM and ANN (E), matching between all the datasets (F), and optimization process of GA (G).

Fig. 3A shows the results of the performance evaluation during training, validation, and testing. The developed network was improved and the best validation performance was 0.055394 of *MSE* values at 7 epochs. *MSE* values were higher when the number of epochs was<3, whereas *MSE* values of training data decreased with the increase of epoch number, which reflected the network over fitting. The model training stopped when the validation error continuously increased 10 times. All in all, the best performance of the model was obtained from the epoch with the lowest validation error (*MSE* values of 0.055394 at 7 epochs). Fig. 3B represents that the data fitting error distribution for training, validation, and testing was within a reasonable good range and was very closed to zero. Fig. 3C displays the training state of the ANN model. The values of gradient, mu, and val fail were 0.0026259, 0.000001, and 3 at 7 epochs, respectively, indicting that the ANN model was well trained. Post training analysis describes that the R values of training, validation, test, and all are 0.9987, 0.95466, 0.95227, and 0.97632, respectively, indicating that there is a good correlation between predicted and actual values (Fig. 3D).

### 3.4 Performance evaluation of RSM and ANN models

By plotting the predicted results generated by the two models (RSM and ANN) and experimental values, a perfected matching was obtained, which means that the two models were designed very well (Fig. 3E). Though, following predictive capacity comparison, it is worth noting that ANN prediction was more accurate than RSM with higher *R*^2^ (0.9620 vs. 0.8253), lower *MSE* (0.0018 vs. 0.0101), *SSE* (0.0266 vs. 0.1515), *AIC* (-46.4027 vs. −20.3075), *RMSE* (0.0421 vs. 0.1005), and *ADD* (1.8124% vs. 2.6153%, Fig. 3F, Table S1). The *R*^2^ values indicated that the variable range of independent variables could explain 82.53% and 96.20 % of the changes in the corresponding COPs yield by RSM and ANN models, respectively. Compared with RSM model, the superiority of ANN model has been previously confirmed by many reports [37], [38]. Therefore, ANN modeling method was selected to optimize the subsequent polysaccharides process in this study.

### 3.5 Optimization of the process

Based on the above analysis results, the GA-ANN method was selected to optimize the extraction process of polysaccharides from *Cornus officinalis* fruit. The corresponding relationship between COPs yield and experimental variables was established by using the 4–10-1 model of ANN, which was used as the fitness function of GA for global optimization. Each component is divided into 30 equal parts to improve the optimization probability and accuracy. Therefore, the substring length (L) of each parameter was 5, and the parameters were combined to form a chromosome with a length (L) of 30. GA randomly generated 20 initial populations, and then obtained the fitness of each individual by using the developed ANN model, and carried out genetic operation on it. Individuals of each generation selected excellent genes with large fitness values through roulette, and then exchanged their excellent genes through two-point crossover (crossover probability of 0.8), and random mutation (mutation probability of 0.05), which generated new genotypes and populations. Subsequently, the new population was evaluated to judge whether it met the algorithm stop criterion. If not, continue to iterate in turn until the individual with the highest fitness appears. The GA-ANN optimization results are presented in Fig. 3G. The population stopped at 70 epochs of iteration. The optimized conditions proposed by GA-ANN are ultrasound power of 355.49 W, extraction temperature of 50.914 ℃, liquid-to-solid ratio of 16.584 mL/g, and extraction time of 38.298 min to obtain the maximum COPs yield (7.66%). The above process parameters are modified based on the actual situation as follows: ultrasound power of 350 W, extraction temperature of 51 ℃, liquid-to-solid ratio of 17 mL/g, and extraction time of 38 min. Under the above parameter combination, the experimental value of COPs yield was 7.85%±0.09%. This result implied that the experimental and the predicted values of COPs yield were in accordance with a 95% confidence interval. Moreover, the results suggested that GA-ANN method was highly suitable for polysaccharides extraction in a nonlinear biological system. The established model had high simulation accuracy and could accurately fit the internal relationship between COPs yield and experimental factors. The predicted results of the model were in good agreement with the actual results.

### 3.6 Characterization

#### 3.6.1 Purification of COPs

**Fig. 4:**
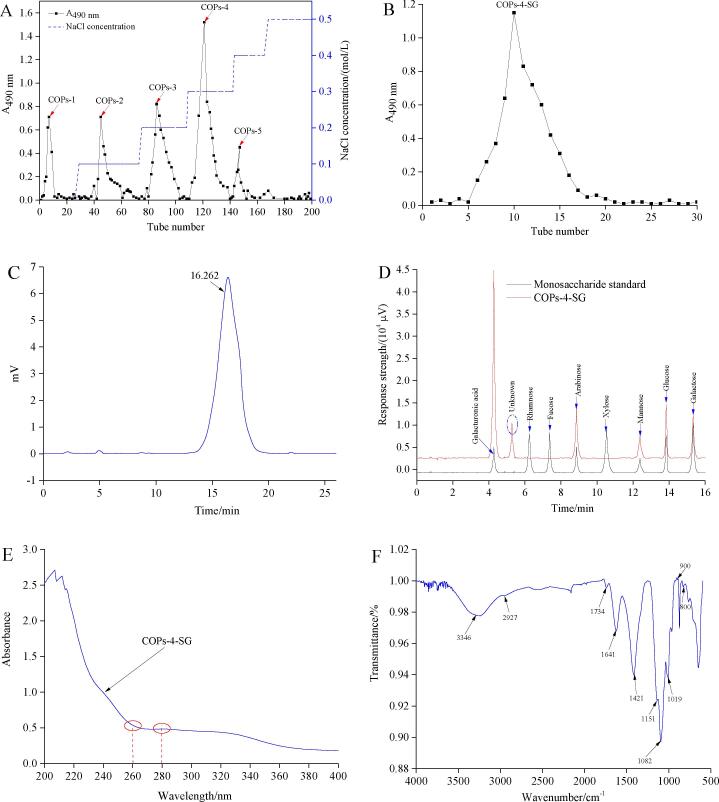
The purification plot of crude COPs on DEAE-52 cellulose column (A); the purification curve of COPs-4 obtained DEAE-52 on Sephadex G-100 column (B); the molecular weight distribution (C), monosaccharide composition (D), UV spectrum (E), and FT*–*IR spectrum of COPs-4-SG (F).

The extract in the lower phase of polysaccharides from *Cornus officinalis* fruit obtained under the optimal extraction parameters was concentrated, precipitated with ethanol (80%), dialyzed, and freeze-dried to obtain the crude COPs. The COPs was separated and purified by DEAE-52 cellulose column, and five fractions (named as COPs-1, COPs-2, COPs-3, COPs-4, and COPs-5) were obtained by elution with deionized water and different concentrations NaCl solutions (0.1, 0.2, 0.3, 0.4, and 0.5 mol/L) (Fig. 4A). Their yields were 28.35%, 13.72%, 21.09%, 17.96%, and 10.14%, respectively. The major fraction (COPs-4) obtained by elution with 0.3 mol/L NaCl solution was further purified by Sephadex G-100 column (1.6 cm × 50 cm), concentrated, and then freeze-dried to obtain a homogeneous polysaccharides fraction (named as COPs-4-SG) with yields of 72.28% (Fig. 4B).

#### 3.6.2 Molecular weight and monosaccharide composition analysis

Molecular weight is an important structural index of polysaccharides, which affects the physicochemical and biological properties of polysaccharides [39]. The average molecular weight of COPs-4-SG was determined by HPGPC method. Fig. 4C shows a single and symmetrical peak, which suggested that COPs-4-SG was a homogeneous polysaccharides. The average molecular weight (M_w_) and number average molecular weight (M_n_) of COPs-4-SG were 33.64 kDa and 31.59 kDa, respectively. This result was lower than that reported in previous studies, FACP1 (M_w_ of 34.5 kDa) a fraction of polysaccharides from the *Cornus officinalis* fruit by 10% NaOH extraction at 4 °C for 4 h and precipitation by ethanol overnight 4 °C [40]. This could be due to the further separation and purification of crude polysaccharides, which removed high molecular weight substances, resulting in the decrease of molecular weight distribution of polysaccharides [41]. The GC was used to further analyze the monosaccharide compositions of COPs-4-SG. Fig. 4D shows the GC charts of hydrolysates of COPs-4-SG. The results indicated that COPs-4-SG was comprised of galacturonic acid, arabinose, mannose, glucose, and galactose in a molar ratio of 34.82:14.19:6.75:13.48:12.26. In addition, COPs-4-SG also contained an unknown monosaccharide, which needed further analysis in the next study. Results show that COPs-4-SG was a heteropolysaccharide with different chemical components. Especially, galacturonic acid was the main monosaccharide. Nevertheless, the results of this study are quite different from that previously reported [42]. This phenomenon might be attributed to different extraction and purification methods, which affected the composition of monosaccharides to a certain extent.

#### 3.6.3 UV–vis and FT–IR spectroscopic analysis

In the ultraviolet absorption spectrum, the sample solution has absorption peaks at the wavelengths of 260 nm and 280 nm, which can verify whether the polysaccharides contain a large amount of protein and nucleic acid [12]. Fig. 4E shows that COPs-4-SG had no obvious absorption peaks at 260 nm and 280 nm, indicating that COPs-4-SG didn’t contain protein, nucleic acid, and anthocyanins. FT*–*IR spectrum of COPs-4-SG were recorded from 4000 to 400 cm^−1^ by using a FT*–*IR spectrometer (Fig. 4F). The FT*–*IR spectrum illustrated a strong and wide stretch vibration of O–H and a weak stretch vibration of saturated C–H at 3346 cm^−1^ and 2927 cm^−1^, respectively[43]. The two peaks at 1641 cm^−1^ and 1421 cm^−1^ are caused by asymmetric and symmetric stretching vibrations of carboxylic acid groups, respectively [44], [45]. This confirmed the presence of uronic acid in COPs-4-SG, which was consistent with the results of monosaccharide composition analysis. A weak peak showed at 1734 cm^−1^ was C = O valent vibration of the O-acetyl group [46]. Three stretching peaks at 1019, 1082, and 1151 cm^−1^ showed the existence of C-O bonds and the pyranose form of sugar [47]. In addition, there are some small peaks in the range of 800 cm^−1^ to 900 cm^−1^, indicating the presence of α- and β- configuration [48].

#### 3.6.4 SEM

**Fig. 5:**
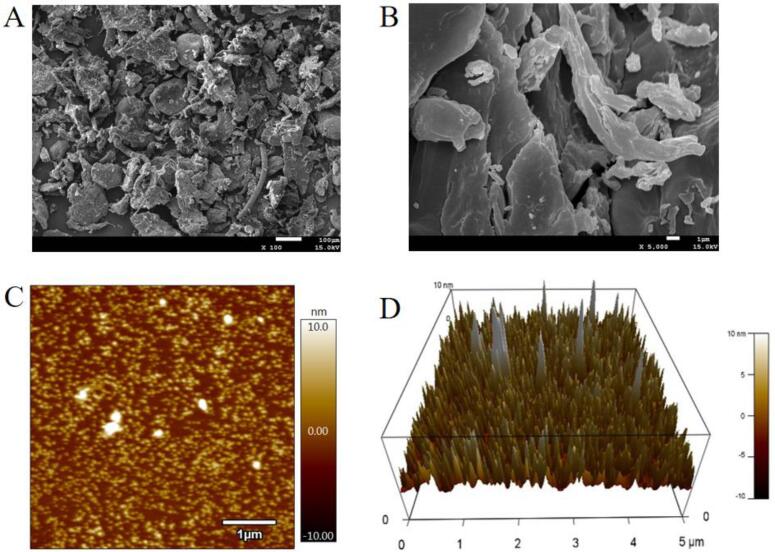
SEM charts (A:100 ×; B:5000 × ), AFM planar image (C), 3D image of AFM in COPs-4-SG (D).

Fig. 5A and 5B show the SEM images of COPs-4-SG at magnifications of 100 × and 5000 ×. COPs-4-SG was mainly composed of irregular and fragmented structures, interspersed with some small nonuniform particles and fragments on the surface. Moreover, some curly morphology could be observed on the surface of COPs-4-SG. This phenomenon may be attributed to the degradation, depolymerization, and reaggregation effects caused by ultrasound treatment [41]. The irregular structure of COPs-4-SG was similar to that of polysaccharides from *Cornus officinalis* seed [42].

#### 3.6.5 AFM

AFM is usually employed to characterize polysaccharides nanostructures and random linear or spherical structure morphology. Fig. 5C and 5D display that COPs-4-SG was mainly composed of spherical lumps. COPs-4-SG formed large lumps indicated that the polysaccharides have undergone molecular aggregation, and the structures of COPs-4-SG were branched and entangled. This phenomenon might be attributed to the fact that the hydroxyl and carboxyl groups of COPs-4-SG could form intimately inter-molecular and intramolecular interactions with each other or with water molecules [49], [50]. Moreover, the mean height of COPs-4-SG was measured using AFM to be about 3.15 nm. This result was significantly higher than that of the single polysaccharides chain (0.1–1.0 nm), further indicating that COPs-4-SG had branches and interweaves with each other [51], whereas the result was lower than that of COPs-4 (4.2 nm). This may be because COPs-4-SG had a smaller molecular weight. Results were consistent with that reported by Zhang et al. (2018)[52].

#### 3.6.6 Congo red test

**Fig. 6:**
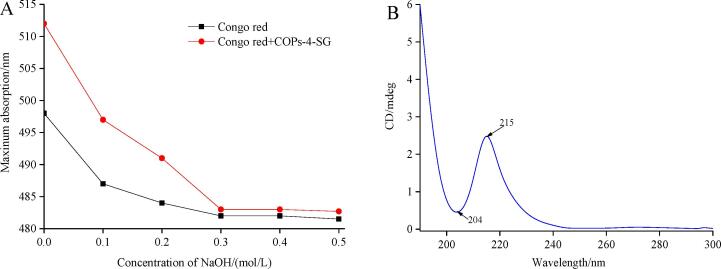
Congo rest plot (A) and CD spectrum (B) of COPs-4-SG.

The polysaccharides contain the triple helical structure, the maximum absorption wavelength (MAW) of the complex formed by Congo red and polysaccharides will have a red shift with the increase of NaOH concentration. If the polysaccharides does not contain the triple helical structure, the change trend of the MAW of UV spectrum is similar to that of Congo red solution. Fig. 6A describes the MAW of Congo-red and Congo-red complex (formed by Congo red and COPs-4-SG) in various NaOH concentrations. The MAW of Congo-red complex was correspondingly decreased with the increase of NaOH concentration. In addition, the specific trend with no red-shift and no remarkable decreasing at higher NaOH concentration. This change trend was similar to the Congo red solution, indicating that COPs-4-SG had non-three helical structure. This result was consistent with the research results of Mao, Hsu, & Hwang, (2007) [53]. Generally, heteropolysaccharides are not easy to form a three spiral structure.

#### 3.6.7 CD

COPs-4-SG was analyzed by CD in the range of 190–300 nm, and the results was displayed in Fig. 6B. COPs-4-SG showed a positive peak at 204 nm, indicating COPs-4-SG had non-three helical structure [12]. This result was consistent with the Congo red test, which proved that COPs-4-SG had non-three helical structure. In addition, COPs-4-SG appeared a maximum positive peak at 215 nm. This might be related to C-O and O–H in COPs-4-SG structure [54].

## 4 Conclusion

Two approaches, RSM and ANN were developed and revealed sufficient reliability in predicting the polysaccharides yield from *Cornus officinalis* fruit. However, ANN prediction was more accurate than RSM. Further, optimization of the UAATPE process was performed by GA-ANN and then obtained the optimum extraction parameters to achieve the highest COPs yield. A homogenous fraction (COPs-4-SG, 33.64 kDa) was isolated from the extracted crude COPs, and the COPs-4-SG contained galacturonic acid, arabinose, mannose, glucose, and galactose. FT*–*IR spectroscopy assay helped to identify the functional groups of COPs-4-SG. AFM observation showed that COPs-4-SG was mainly composed of spherical lumps. SEM results displayed that COPs-4-SG included irregular and fragmented structures, interspersed with some small nonuniform particles and fragments on the surface. The Congo red and CD tests described that COPs-4-SG had non-three helical structure. This study provides necessary information for the extraction, purification, and process optimization of polysaccharides from *Cornus officinalis* fruit. However, the relationship between the structure and activities of polysaccharides still needs to be further explored.

### CRediT authorship contribution statement

**Jiaqi Tan:** Data curation, Visualization, Writing – original draft, Writing – review & editing. **Pengshan Cui:** Methodology, Formal analysis, Writing – review & editing. **Shaoqin Ge:** Formal analysis, Software. **Xu Cai:** Formal analysis, Software. **Qian Li:** . **Hongkun Xue:** Conceptualization, Software, Writing – original draft, Resources, Supervision, Writing – review & editing.

## Declaration of Competing Interest

The authors declare that they have no known competing financial interests or personal relationships that could have appeared to influence the work reported in this paper.
