# Supplementary material for: Ultrasound assisted aqueous two-phase extraction of polysaccharides from Cornus officinalis fruit: Modeling, optimization, purification, and characterization
Source: Ultrason Sonochem. 2022 Mar 1;84:105966. doi: 10.1016/j.ultsonch.2022.105966 (PMC8897718; doi:10.1016/j.ultsonch.2022.105966)
Supplement: Supplementary data 1 [file mmc1.docx]

**Table of Contents**

| CONTENTS | 1 |
| --- | --- |
| **Fig. S1** Influence of the number of hidden layer neurons on *MSE* | 2 |
| **Table S1** Calculated predictive capacity of RSM and ANN models for COPs yield | 3 |

Fig. S1

Table S1

| Calculated predictive capacity | RSM | ANN |
| --- | --- | --- |
| *R*^2^ | 0.8253 | 0.9620 |
| *MSE* | 0.0101 | 0.0018 |
| *RMSE* | 0.1005 | 0.0421 |
| *SSE* | 0.1515 | 0.0266 |
| *AIC* | -20.3075 | -46.4027 |
| *ADD* (%) | 2.6153 | 1.8124 |
